# Intrathecal Injection of Dual Zipper Kinase shRNA Alleviating the Neuropathic Pain in a Chronic Constrictive Nerve Injury Model

**DOI:** 10.3390/ijms19082421

**Published:** 2018-08-16

**Authors:** Meei-Ling Sheu, Chien-Yi Chiang, Hong-Lin Su, Chun-Jung Chen, Jason Sheehan, Hung-Chuan Pan

**Affiliations:** 1 Institute of Biomedical Science, National Chung-Hsing University, Taichung 40244, Taiwan; mlsheu@nchu.edu.tw; 2 Department of Neurosurgery, Taichung Veterans General Hospital, Taichung 40754, Taiwan; evils1019@hotmail.com; 3 Department Life Sciences, Agriculture Biotechnology Center, National Chung Hsing University, Taichung 40244, Taiwan; suhonglin@gmail.com; 4 Department of Medical Research, Taichung Veterans General Hospital, Taichung 40754, Taiwan; cjchen@vghtc.gov.tw; 5 Department of Neurosurgery, University of Virginia, Charlottesville, VA 22901, USA; jps2f@hscmail.mcc.virginia.edu; 6 Faculty of Medicine, School of Medicine, National Yang-Ming University, Taipei 11257, Taiwan

**Keywords:** dual zipper kinase, chronic constrictive injury, neuropathic pain

## Abstract

Dual leucine zipper kinase (DLK) is a member of mitogen-activated protein kinase kinase kinase (MAP3K) family mainly involved in neuronal degeneration. However, the role of DLK signaling in the neuropathic pain has not yet been fully determined. Chronic constrictive injury (CCI) was conducted by four 3-0 chromic gut ligatures loosely ligated around the sciatic nerve. Escalated DLK expression over the dorsal root ganglion was observed from one to four rings of CCI. Remarkable expression of DLK was observed in primary dorsal root ganglion cells culture subjected to electrical stimulation and attenuated by DLK short hairpin RNA (shRNA) treatment. Intrathecal injection of DLK shRNA attenuates the expression of DLK over the dorsal root ganglion and hippocampus neurons and increased the threshold of mechanical allodynia and decreased thermal hyperalgesia. In CatWalk gait analysis, significant decreases of print area, maximum contact maximum intensity, stand phase, single stance, and regular index by CCI were alleviated by the DLK shRNA administration. In conclusion, the expression of DLK was up-regulated in chronic constrictive injury and attenuated by the administration of DLK shRNA, which paralleled the improvement of neurobehavior of neuropathic pain. The modulation of DLK expression is a potential clinic treatment option for neuropathic pain.

## 1. Introduction

Neuropathic pain is well-known for a damage involved in the somatosensory system, and is usually defined as pain initiated or caused by a primary lesion or dysfunction of the nervous system [1,2]. A high proportion of the general population, up to 8%, will experience chronic pain associated with neuropathic features, which significantly affects their lives psychologically, physically, and socially. This pain usually poorly responds to traditional analgesics such as anti-inflammatory and opiate drugs [1]. Therefore, it remains quite a large challenge for both scientists and clinicians.

In general, nerve injury and spinal cord damage induces an extreme activation of mitogen-activated protein kinase (MAPK) in glial cells of the spinal cord and on some occasions, peripheral nerve injury also activates the downstream cascade of p38 and extracellular signal-regulated kinases (ERK) in spinal microglia [3,4,5,6]. For glial cells, MAPK activation is essential for the maintenance and development of neuropathic pain, and this activation plays a role in the neuronal related mechanisms of chronic pain and hypersensivity [7,8,9]. As a mixed lineage kinase from the kinases of mitogen-activated protein kinase kinase kinases (MAP3K) for c-Jun N-terminal kinases (JNKs), dual leucine zipper bearing kinase (DLK) is worth noting [10,11,12]. However, DLK is most abundantly expressed in nerve tissue especially distributed in neurons [13,14,15]. Injury to the axon leads to activation of DLK downstream targets including mitogen-activated protein kinase (MAPK)/c-Jun N-terminal kinase (JNK) [16]. In severed axons of Drosophila and mice, dual leucine kinase (DLK) can promote degeneration, and the resulting JNK target also leads to local degeneration of axons as they proceed down the path to degeneration [17]. The pathological feature of peripheral human neuropathy is very similar to those seen in nerve Wallerian degeneration [18]. Thus, the modulation of nerve crush associated degeneration by DLK seems to be a potential role in the alleviation of neuropathic pain.

DLK is regarded as more attractive than other approaches aimed at inhibition of MAPKs signaling in the treatment of neuropathic pain. First, DLK expression is predominantly distributed in neurons and reasonably provides a method to target neuronal dysfunction in central nervous system disease, in contrast to many other components of the JNK signaling pathway mainly distributed in the glia cells [15,19,20]. Second, destruction of DLK signaling in nervous system is well tolerated [21]. Third, DLK regulated the broad stress response upon neuron injury and was rapidly rescued by the DLK inhibitors [22], which potentially attenuate a range of downstream signaling events in the context of the neuron degeneration process [23]. Based on the potential role for DLK signaling involved in multiple disease models driven by various mechanisms, inhibition of DLK could possess a broad therapeutic potential for neuropathic pain.

The aim of this study was to elucidate the potential role of intrathecal injection of DLK short hairpin RNA (shRNA) to assess the neurobehavior and histomorphology alteration of somatosensory neuron in a chronic constrictive nerve injury animal model. This study showed that escalated expression of DLK in dorsal root ganglion and hippocampus neurons subjected to the severity of nerve constrictive injury, and the alleviation of neuroinflammation and improvement of neurobehavior were shown by the intrathecal injection of DLK ShRNA.

## 2. Results

### 2.1. Escalated Expression of DLK Expression in Dorsal Root Ganglion Cells Subjected to Different Intensities of Nerve Injury

**Figure 1:**
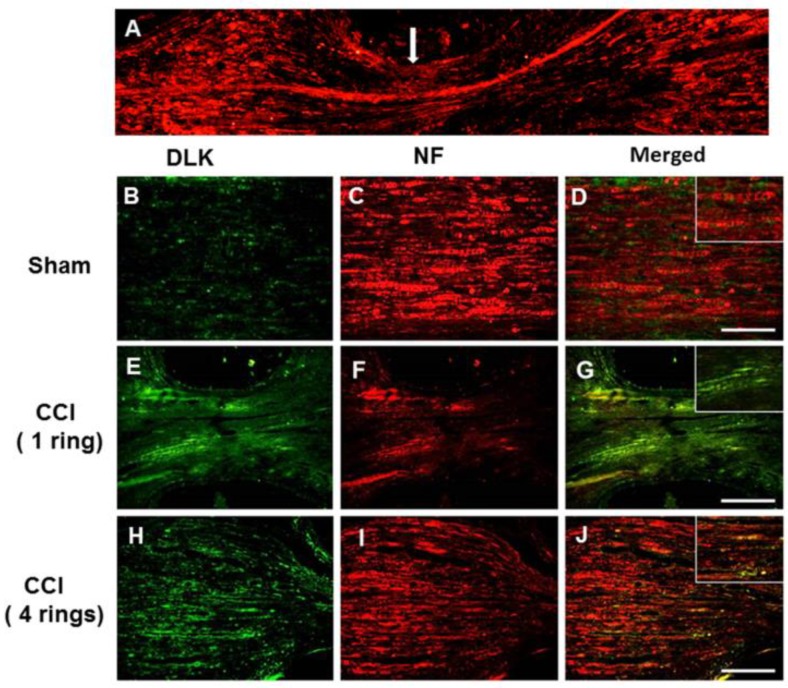
Representative dual leucine zipper kinase (DLK) and neurofilament (NF) expression over the injury site of the nerve in chronic constrictive injury (CCI) animals four weeks after injury. (**A**) Distribution of DLK distal and proximal to the point of CCI injury. (**B**) Distribution of DLK in the proximal site of the sham group. (**C**) Co-localized with Neurofilament. (**D**) Merged imaging fusion of (**B**,**C**), right upper box indicated the imaging amplification. (**E**) Distribution of DLK in the proximal site of one ring CCI. (**F**) Co-localized with Neurofilament. (**G**) Merged imaging fusion of E and F, right upper box indicated imaging amplification. (**H**) Distribution of DLK in the proximal site of four rings CCI. (**I**) Co-localized with Neurofilament. (**J**) Merged imaging fusion of (**H**,**I**), right upper box indicated imaging amplification. Bar length = 200 μm; Arrow indicated the nerve crushed region.

**Figure 2:**
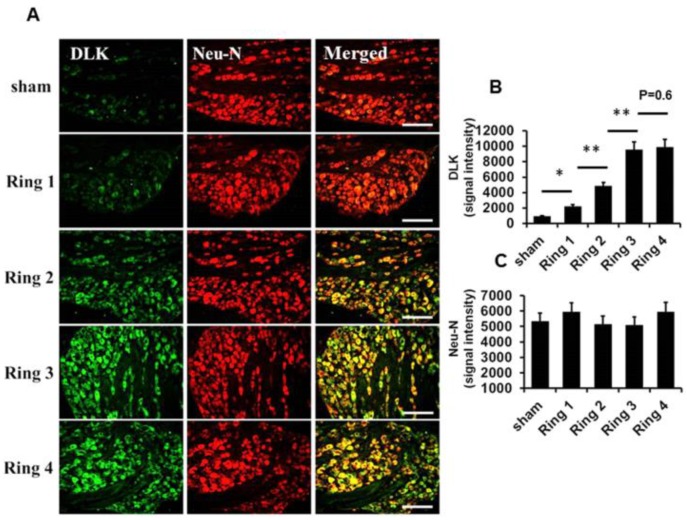
Depiction of DLK expression over the dorsal root ganglia cells subjected to the escalating numbers of ligature rings from one to four in animals four weeks after CCI. (**A**) Illustration of DLK expression over the dorsal root ganglion cells co-localized with Neu-N related to the escalated number of nerve ligations from one to four rings. (**B**,**C**) Quantitative analysis for expression level of DLK and Neu-N in the dorsal root ganglia cells. Bar length= 200 μm; * *p* < 0.05; ** *p* < 0.01.

The characteristic findings of increased intensity of chronic constrictive injury (CCI) paralleled the severity of inflammatory response in the dorsal root ganglion cells published in our group [24]. In general, DLK is most abundantly expressed in nerve tissue and is especially distributed in neurons [13]. First, we assessed the possibility of severity of nerve injury related to the expression of DLK in damaged nerve. In the CCI model with four ring ligature, the DLK was distributed distally and proximally of the site on the injured side (Figure 1A). The progressively increased expressions of DLK were shown from the sham, one ring to four rings catgut ligature, and the expression mostly co-localized with the neuron marker of neurofilament (Figure 1B–J). In addition, the dorsal ganglion cell was the hall marker for the assessment of severity of neuropathic pain. The escalated expression of DLK in dorsal root ganglion cells was noted from sham to four ring ligatures, and these expressions also co-localized with the expression of the neuron marker of Neu-N (Figure 2A). The statistical analysis showed the significant escalation of DLK from sham to four rings ligature (Figure 2B,C). Thus, the significant expression of DLK in axon and dorsal root ganglion cells was highly correlated with the severity of nerve injury. According to the guidelines of NC3Rs ARRIVE (Animal Research: Reporting in vivo Experiments), to reduce numbers of animals used in the experimental study, the following animal study of CCI used four rings ligature as the CCI model for the following investigation.

### 2.2. Construction of DLK shRNA for the Assessment of Inhibition Effects

**Figure 3:**
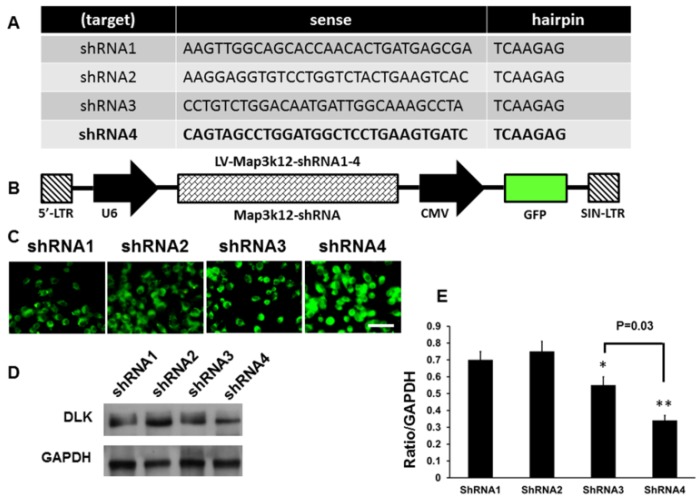
Construction of short hairpin RNA (shRNA) of DLK for the assessment of the inhibitory effect after intrathecal injection in fours rings CCI model. (**A**) Four specified sequence of shRNA targeting DLK were searched from OriGene web site (www.OriGene.com/shRNA). (**B**) Schematic presentation of the plasmid vector and encoded GFP protein and DLK shRNA sequence. (**C**) The representative of 293T mammalian cells infected by the different sequence of DLK shRNA. (**D**) Representative of Western blot analysis of DLK expression in dorsal root ganglion cells subjected to intrathecal injection of DLK shRNA. (**E**) Quantitative analysis of Western blot analysis of DLK in dorsal root ganglion cells. Bar length = 200 μm; * *p* < 0.05; ** *p* < 0.01. GAPDH: glyceraldehyde-3-phosphate dehydrogenase; CMV: Cytomegalovirus; SIN-LTR: Self-Inactivating- Long terminal repeat.

The four specified sequences of shRNA are illustrated in Figure 3A, and the schematic presentation of the plasmid vector and encoded GFP protein and DLK shRNA sequence is shown in Figure 3B. The successful transfections of 293T mammalian cells were demonstrated in Figure 3C. The assessment in the power of knock down of DLK expression in the dorsal root ganglion cells in CCI animals showed the most significant inhibition of DLK by sequence 4 (Figure 3D,E). Hence, we used the specified sequence 4 DLK shRNA in the following cell culture and animal study.

### 2.3. Expression of DLK in Primary Dorsal Root Ganglion Cells Culture Subjected to High-Frequency Electrical Stimulation Abolished by DLK shRNA

**Figure 4:**
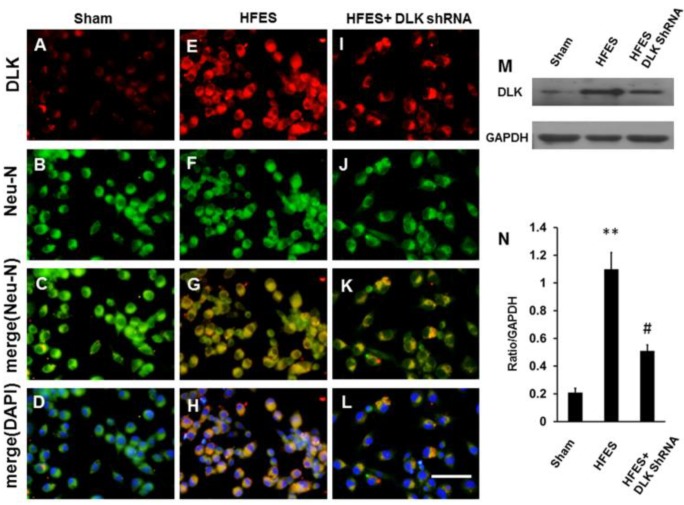
Illustration of DLK expression in dorsal root ganglion cells culture subjected to high frequency electrical stimulation (HFES) and DLK shRNA treatment. (**A**) Expression of DLK in dorsal root ganglion cells culture. (**B**) Co-localization with Neu-N. (**C**) Merged imaging of (**A**,**B**). (**D**) Merged imaging of (**A**–**C**). (**E**) Expression of DLK in dorsal root ganglion cells culture by high frequency electrical stimulation. (**F**) Co-localization with Neu-N. (**G**) Merged imaging of (**E**,**F**). (**H**) Merged imaging of (**E**,**F**,**G**). (**I**) Expression of DLK in dorsal root ganglion cells culture by high frequency electrical stimulation followed by DLK shRNA. (**J**) Co-localization with Neu-N. (**K**) Merged imaging of (**I**,**J**). (**L**) Merged of (**I**,**J**,**K**). (**M**) Illustration of Western blot imaging (**N**). Quantitative analysis of Western blot analysis. ** *p* < 0.001 indicated high frequency electrical stimulation group relative to sham; # *p* < 0.05 indicated the DLK shRNA treated group relative to high frequency electrical stimulation group; Bar length = 200 μm.

The mimicking of neuropathic pain in the dorsal root ganglion cells by high-frequency electrical stimulation was used in our group [25]. The high expression of DLK was shown when subjected to high-frequency electrical stimulation, and this effect was abolished by the DLK shRNA administration. These expressions were almost predominately distributed in the nucleus of dorsal root ganglion cells (Figure 4A–L). A representative Western blot analysis also showed the same trends as the immunofluorescent staining (Figure 4M). The statistical analysis showed significant abolishment of DLK expression in dorsal root ganglion cells induced by electrical stimulation after the administration of DLK shRNA (Figure 4N).

### 2.4. Improvement of Neurobehavior of CCI by the Intrathecal Injection of DLK shRNA

**Figure 5:**
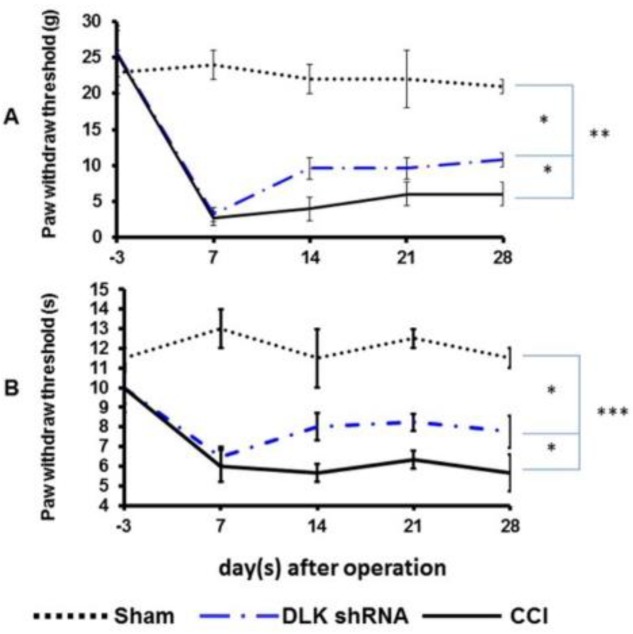
Representative images of mechanical allodynia and thermal hyperalgesia treated by DLK shRNA administrated 24 h after CCI. (**A**) Plot of mechanical allodynia in different treatment groups in gm related to different time frame. (**B**) Plot of thermal hyperalgesia in different treatment groups related to different time frame. * *p* < 0.05; ** *p* < 0.01; *** *p* < 0.001.

The von Frey and thermal tests were the standard assessments for the neuropathic pain. The significantly decreased paw mean withdraw threshold was noted from 25 to 5 gm three days before and seven days after CCI, and these effects lasted 28 days. The intrathecal injection of DLK shRNA significantly attenuated this response at day 14 and maintained the response to 28 days (Figure 5A). The mean latency of thermal withdraw was 11 s before injury and then decreased to 6 s at day 7 and maintained the threshold up to 28 days in the control group. The intrathecal injection of DLK shRNA significantly increased the threshold at day 14 and maintained the effect to 28 days (Figure 5B).

**Figure 6:**
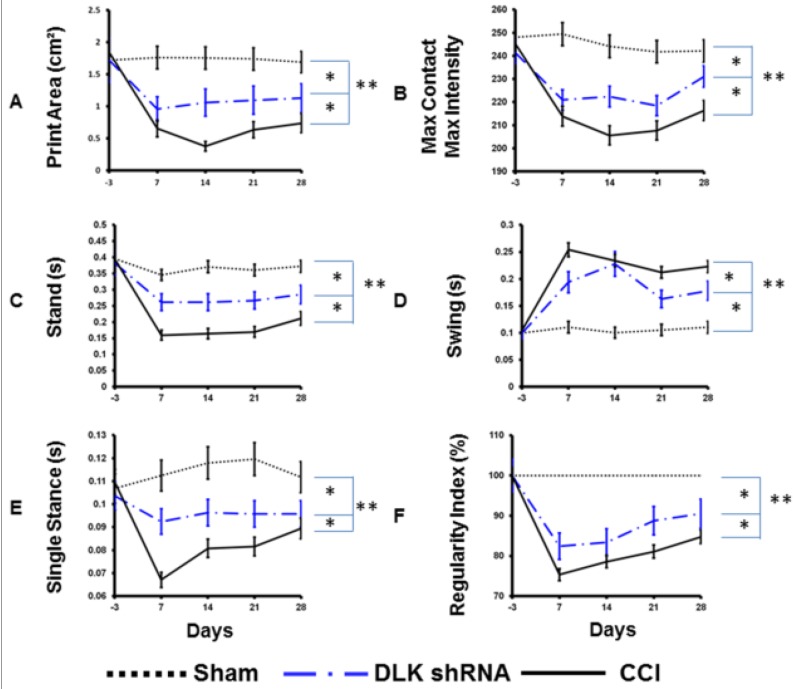
Representative data of the CatWalk XT parameters in CCI animals treated by DLK shRNA administrated 24 h after CCI. (**A**) Illustration of intensity of printed area in various treatment groups related to different time frames after operation. (**B**) Illustration of maximum contact maximum intensity in the various treatment groups related to different time frames after operation. (**C**) Illustration of stand phase in various treatment groups related to different time frames after operation. (**D**) Illustration of swing phase in various treatment groups related to different time frames. (**E**) Illustration of single stance in various treatment groups related to different time frames. (**F**) Illustration of regular index in the various treatment groups related to different time frames. * *p* < 0.05; ** *p* < 0.01.

Catwalk gait analysis showed the static and dynamic power in the assessment of neuropathic pain. The CCI caused a significant decrease of print intensity and maximum contact and maximum intensity, and these decreases were attenuated by the intrathecal injection of DLK shRNA of (Figure 6A,B). The remarkably decreased stand phase was observed after CCI, and the trend was mitigated by the DLK shRNA (Figure 6C). The reciprocal effect of decreased swing phase by the administration of DLK shRNA was noted (Figure 6D). The parameters of single stance showed a very similar result to data of the stance phase (Figure 6E). The regularity index denoted the coordinated effect of four limbs. CCI caused the dropping effect of regularity index from 100% to 80% at 7 days after injury and lasting to 28 days, and the return of RI was restored by the DLK shRNA starting at day 7 and postponed the effect to 28 days (Figure 6F).

### 2.5. Attenuation of DLK over the Dorsal Root Ganglion and Hippocampus Subjected to Intrathecal Injection of shRNA of DLK

**Figure 7:**
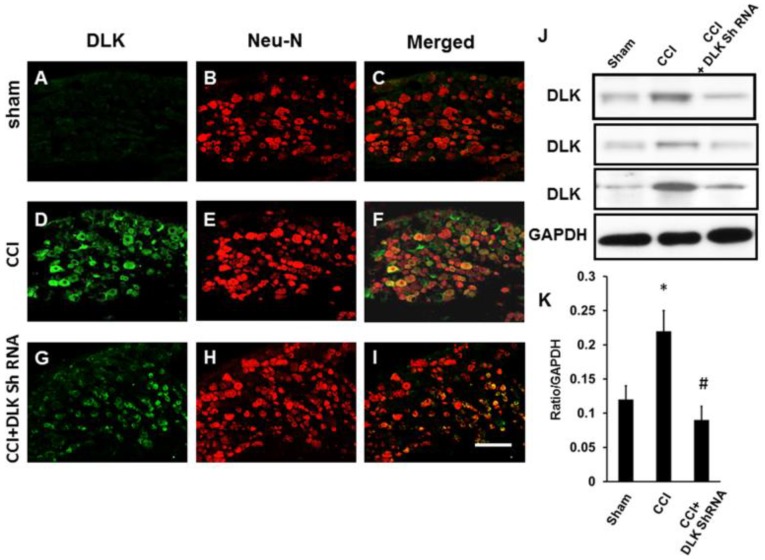
Illustration of DLK in dorsal root ganglion cells in CCI animals subjected to intrathecal injection of DLK shRNA. (**A**) Expression of DLK in dorsal root ganglion cells in the sham group. (**B**) Co-localization with Neu-N. (**C**) Merged imaging of (**A**,**B**). (**D**) Expression of DLK in dorsal root ganglion cells in the four rings CCI group. (**E**) Co-localization with Neu-N. (**F**) Merged imaging of (**D**,**E**). (**G**) Expression of DLK in dorsal root ganglion cells in the four rings CCI group treated by intrathecal administration of shRNA of DLK. (**H**) Co-localization with Neu-N. (**I**) Merged of (**G**,**H**). (**J**) Illustration of Western blot imaging. (**K**) Quantitative analysis of Western blot analysis. * *p* < 0.05 indicated four rings CCI relative to sham; # *p* < 0.05 indicated the shRNA of DLK relative to four rings CCI group. Bar length = 200 μm.

**Figure 8:**
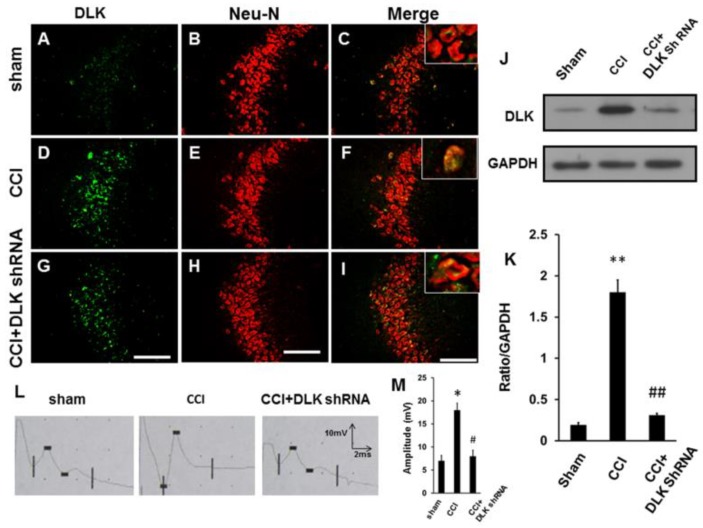
Alteration of DLK expression in the hippocampus and evoked potential subjected to intrathecal injection of DLK shRNA. (**A**) Expression of DLK in the hippocampus in the sham group. (**B**) Co-localization with Neu-N. (**C**) Merged imaging of (**A**,**B**), right upper box indicated the imaging amplification. (**D**) Expression of DLK in hippocampus in the four rings CCI group. (**E**) Co-localization with Neu-N. (**F**) Merged imaging of (**D**,**E**), right upper box indicates the imaging amplification. (**G**) Expression of DLK in the hippocampus in the four rings CCI group treated by intrathecal administration of DLK shRNA. (**H**) Co-localization with Neu-N. (**I**) Merged imaging of G and H, right upper box indicates the imaging amplification. (**J**) Illustration of Western blot imaging. (**K**) Quantitative analysis of Western blot analysis. (**L**) Illustration of the amplitude of somatosensory evoked potential in different treated group. (**M**) Quantitative analysis of the amplitude of somatosensory evoked potential in different treatment groups. * *p* < 0.05, ** *p* < 0.01 indicated four rings CCI relative to sham; # *p* < 0.05, ## *p* < 0.01 indicated the shRNA of DLK relative to four rings CCI group. Bar length = 200 μm.

The DLK was up-regulated in dorsal root ganglion cells subjected to CCI injury (Figure 7A–F). The intrathecal injection of DLK shRNA attenuated the response (Figure 7G–I). The Western blot analysis also showed the same trend (Figure 7J), and the significantly decreased expression of DLK was shown in the quantitative analysis (Figure 7K). Furthermore, the increased expression of DLK was noted over the hippocampus after CCI (Figure 8A–F). This expression was attenuated by the intrathecal injection of DLK shRNA (Figure 8G–I). The Western blot analysis also showed the same trend (Figure 8J–K). Furthermore, the amplitude of the somatosensory evoked potential was elucidated by the CCI, and this effect was attenuated by the administration of intrathecal injection of DLK shRNA (Figure 8L,M).

## 3. Discussion

DLK has been known to be distributed in axons and synaptic terminals for two decades [11,15], and it promotes degeneration of severed axons in dorsal root ganglion cells and knockdown DLK gene attenuates the nerve degeneration [17]. The pathological feature of peripheral human neuropathy is very similar to that seen in axotomy-induced Wallerian degeneration [18]. This study showed the increased expression of DLK in nerves, dorsal root ganglion cells, and hippocampus neurons related to the severity of nerve injury. The attenuation of DLK by intrathecal shRNA of DLK was in line with the improvement of neurological outcome. Thus, the target of DLK seems a promising tool to overcome the Wallerian degeneration induced neuropathic pain.

The DLK signaling pathway regulates several aspects of neural development ranging from axon growth and neuronal migration to apoptosis and axon degeneration in different model organisms [13,26]. During the development in mice, DLK protein was localized in the brain, spinal cord, and sensory ganglia [13,14]. In the oxidative stress and in a scarcity of trophic factors, the activation of DLK-dependent signaling cascades caused rapid neuron degeneration during the development of embryos [27,28]. In the setting of genetic alterations, DLK disruption can lead to reduction in activation of JNK and result in reduced phosphorylation of JNK targets. This can result in defects within neuronal migration and the impairment of axonal tract development in the corpus callosum and anterior commissure [13]. In this study, nerve constrictive injury caused the escalated expression of DLK in somatosensory neurons, which are down-regulated by shRNA, and the down-regulation by shRNA paralleled the improvement of neuropathic pain. The modulation of DLK in the CCI model seems to be consistent with the previous literature on axotomy-induced Wallerian degeneration by DLK and was rescued by gene knockdown [17].

With loose chromic ligatures on the rat’s sciatic nerve in place, behavior that was analogous to human neuropathic pain resulted [29]. The intensity of nerve injury was correlated to the inflammatory response over the dorsal root ganglion and somatosensory cortex, as well as the severity of nerve degeneration and the works of [24,30]. Upon nerve axotomy, the injured nerve showed up-regulation of DLK over the neuron and dorsal root ganglion and decreased nerve degeneration was attenuated by the DLK gene knockdown [13,14,17]. This study showed the DLK expression in the dorsal root ganglion and somatosensory cortex was in line with the alteration of neurobehavior. The intrathecal injection of DLK shRNA attenuated the DLK expression in the aforementioned tissues and also relieved the pain behaviors. The result further confirms the regulation of DLK in somatosensory neuron contributed to the development of neuropathic pain. These results can be a hypothesis generating for future research directions, as we will highlight. 

Persistent chronic pain not only caused sensory dysfunction, but also produced various brain disorders, which led to a high level of cortical or subcortical dysfunction [31]. The physiology and structural remodeling the learning circulatory of the hippocampus were noted when subjected to stress or chronic pain [32]. In our previous publication, the severity of chronic constriction injury was highly correlated with the intensity of inflammatory response in the hippocampus and attenuation of the neuropathic pain caused the decreased expression of the inflammatory response in the hippocampus [24,30]. As known, deletion of DLK in the neuron is essential for the improvement of the neuropathic pain or attenuation of nerve degeneration after nerve injury, but there was no mention of DLK distribution in the hippocampus [18,33]. In this study, we found that chronic constriction injury caused the significant expression of DLK from nerve to dorsal root ganglion and attenuated by the intrathecal injection of DLK shRNA, which paralleled with previous reports [13,14,17,33]. Thus, the significantly high expression of DLK in the hippocampus in this study was postulated because of remodeling of the learning circuit when animals were subjected to neuropathic pain. 

There may be questioning of the fact that we did not conduct the intrathecal injection of DLK shRNA in the normal animal for the assessment of the collateral effects such as the abnormal behaviors. In this study, we only conduct the intrathecal injection of DLK shRNA in the CCI animal compared with phosphate-buffered saline (PBS) injection to assess the alteration of neurobehavior. Therefore, the DLK shRNA in the normal animals should be used as the control in the following study. 

## 4. Materials and Methods

### 4.1. Assessment of High Frequency Electrically Stimulated Dorsal Root Ganglion Primary Culture Cells Treated by DLK shRNA

Dorsal root ganglia (DRG) cells were harvested from embryonic day 14–15 rats, and the method had been published in our previous study [30]. In brief, the embryonic DRG cells were dissected and then were put in HBSS (Hanks’ balanced salt solution; Invitrogen, Waltham, MA, USA), which contained 5 mg/mL dispase (Roche Applied Science, Penzberg, Germany), for 5 min at 37 °C, and subjected to centrifuge for 5 min at 3000–5000 rpm at room temperature. The DRG cells were diluted and suspended in neurobasal medium to achieve the density of 2 × 10^4^ cells/well in 24-well plates. Finally, the well plate was placed into a 37 °C, 5% CO_2_ incubator and the cells were left undisturbed for the following experiment. The cells were subjected to high frequency electrical stimulation for duration of 30 min at frequencies of 100 Hz with 50 mA [25]. The cells were then subjected to immunohistochemical staining and Western blot analysis.

### 4.2. Construction of DLK shRNA

The four unique 29-mer shRNA constructs in lentiviral GFP vector of DLK/Map3k12 Rat shRNA plasmid (Locus ID 25579, CAT#: TL709656, OriGene Technologies, Inc., Rockville, MD, USA) was purchased and certificate by OriGene Technologies, Inc. (Rockville, MD, USA). In brief, the search of the DLK target gene on the OriGene web site (Available online: www.OriGene.com/shRNA) was conducted. Finally, there were four sequences for construction, including the following:
shRNA1: AAGTTGGCAGCACCAACACTGATGAGCGA;shRNA2: AAGGAGGTGTCCTGGTCTACTGAAGTCAC;shRNA3: CCTGTCTGGACAATGATTGGCAAAGCCTA;shRNA4: CAGTAGCCTGGATGGCTCCTGAAGTGATC


OriGene guarantees that the sequences in the shRNA expression cassettes are verified to correspond to the target gene with 100% identity. Each shRNA vector is cloned in pGFP-C shLenti plasmid under a U6 promoter and antibiotic selection by chloramphenicol (34 mg/mL) for mammalian cell expression. The product is suitable for transient and long term silencing through RNAi mechanism. Five micrograms of purified plasmid for each vector is dried in a vial ready to be reconstituting for transformation and amplification. All plasmid products with shRNA expression cassettes have been isolated from single colonies. The plasmids were purified using OriGene’s ion exchange plasmid purification system (PowerPre. HP Midiprep Kits with Prefilter NP 100024). They were examined on agarose gel to ensure the presence and appropriate quality of the plasmids.

### 4.3. Animal Model of CCI

Sprague–Dawley rats weighing 250–300 g were used in this study; permission for their use was approved by the Institutional Animal Care and Use Committee (IACUC) of Taichung Veterans General Hospital. The method of CCI model has been published in our group [24]. In brief, anesthesia of animals was induced with 4% isoflurane and maintained with 1–2% isoflurane. These animals were subjected to one to four 3-0 chromic gut ligatures loosely ligated around the left sciatic nerve according to different experimental requirement. The animals were allocated to three groups including the sham (*n* = 6), CCI (*n* = 6), and CCI + DLK shRNA (*n* = 6). The method of delivery and the amount of shRNA had been described previously [34]. In brief, a total volume of 10 μL (2 × 10^12^ vector genomes copies per microliter) or PBS were injected slowly over a 2-min period over the L4–5 spinal level 24 h after CCI. After operation, the animals were kept in a temperature-controlled environment at 20 °C, and were exposed to alternating light and dark cycles of 12 h. All animals were treated and cared for in accordance with the guidelines recommended by IACUC of Taichung Veterans General Hospital (La-1041302) (17 June 2015) and NC3Rs ARRIVE (Animal Research: Reporting in vivo Experiments).

### 4.4. Mechanical Allodynia and Thermal Hyperalgesia

The method of assessment of the thermal hyperalgesia and mechanical allodynia method had been described in our group [24,30]. A rat was placed on a customized acrylic chamber with dimensions of 20 cm × 20 cm × 20 cm and contained 2-mm-diameter holes in a 5-mm grid of perpendicular rows throughout the entire area of the platform. The assessment of mechanical allodynia applied a von Frey hair (touch-test sensory evaluator) (North Coast Medical, Gilroy, CA, USA) to the hind paw five times at 5-s intervals. The value of mechanical withdraw in gm was defined as the withdrawn value from a particular hair either four or five times out of the five applications. Thermal hyperalgesia was assessed by hot-plate test (Technical & Scientific Equipment GmbH, Thuringia, Germany) according to our previous procedure [30]. The paw withdrawal latency was defined as the interval from the rat touching the 52 °C hot plate to withdraw the paw and 20 s was used a maximal cutoff time to avoid the thermal injury.

### 4.5. CatWalk Automated Quantitative Gait Analysis

The CatWalk (Noldus, Wageningen, Netherlands) gait analysis has been described in our previous study for assessment of gait alteration [35]. In short, the CatWalk XT system comes with a high-speed digital camera, which transforms each scene into a digital image. The digital images are transferred to a computer through an Ethernet connection. The brightness of a pixel was the amount of light received from such an area by the camera. The data from the CatWalk XT system was quantitatively analyzed including the following parameters: maximum contact maximum intensity (maximum intensity at the maximum contact of a paw), print area, duration of swing and stance phases, steps sequence distribution, and regularity index.

### 4.6. Somatosensory Evoked Potential

The assessment of somatosensory evoked potential has been published in our group [24]. After the rats were anesthetized, bilateral recording electrode were threaded into the dural surface of the somatosensory area (3 mm lateral and 2 mm posterior to the bregma) one day before euthanasia. The evoked potential was determined by a threaded electrode over the skull and a reference needle 20 mm from the recorded electrode. Electrical stimulation was applied at the point of 1 cm above the injury site with the stimulation intensity of 20 mA and filtration range of 20–2000 Hz. The data were presented as the ratio of the injured side divided by the normal side to reduce the effect of anesthesia.

### 4.7. Western Blot Analysis

The distal end of the injury nerve, dorsal root ganglion cell, and hippocampus were obtained four weeks after CCI, and the proteins were extracted. Proteins (50 μg) were resolved by SDS (sodium dodecyl sulfate)-polyacrylamide gel electrophoresis and transferred onto blotting membranes. After blocking the membranes with non-fat milk, they were incubated with neurofilament (1:200 dilutions) (Merck Millipore, Burlington, MA, USA), Neu-N (1:500) (Merck Millipore, Burlington, MA, USA), MAP3K (DLK) (1:200 dilution) (Novus, Littleton, CO, USA), and GAPDH (glyceraldehyde-3-phosphate dehydrogenase) (1:2000 dilutions) (Santa Cruz Biotechnology, Dallas, TX, USA) antibodies overnight at 4 °C. The determination method had been published in our group. The similar detection method had been published in our group [30]. 

### 4.8. Immunohistochemical ANALYSIS

Animals were anesthetized four weeks after CCI and then perfused with phosphate-buffered saline (PBS), followed by a fixative solution containing 4% paraformaldehyde. Hippocampus, dorsal root ganglia (bilateral L-4 to L-6 dorsal root ganglia), and sciatic nerves were resected and placed in 4% paraformaldehyde for 4 h, and then transferred to 30% sucrose at 4 °C overnight. The samples were subsequently embedded in Tissue-Tek O.C.T. Compound (Sakura) and rapidly frozen. Serial 8-μm thick sections of sciatic nerve, dorsal root ganglion, and brain were cut on a cryostat and mounted on Superfrost Plus slides (MenzelGlaser) (Thermo Scientific, Waltham, MA, USA). The sections were then subjected to immunohistochemical examination with antibodies against MAP3K (DLK) (1:200 dilution) (Novus, Littleton, CO, USA), neurofilament (1:500 dilutions) (Merck Millipore, Burlington, MA, USA), and Neu-N (1:500) (Merck Millipore, Burlington, MA, USA) for the detection of inflammatory response and the identity of inflammatory cells. The immunoreactive signals were visualized by using goat anti–mouse Immunoglobulin G (fluorescein isothiocyanate) (1:200 dilutions) (Jackson Immuno Research, West Grove, PA, USA) and anti-mouse Immunoglobulin G (rhodamine) (1:200 dilution) (Jackson Immuno Research, West Grove, PA, USA). Six tissue specimens in each group were cut into 8-μm thick sections and stained with antibody. The immunoreactive signals were observed using AF 488 donkey anti-mouse IgG and AF594 donkey anti-rabbit (1:200 dilutions) (Invitrogen, Carlsbad, CA, USA), and were then viewed using an Olympus BX40 Research Microscope.

### 4.9. Statistical Analysis

Data are shown as the mean ± SE (standard error). The CatWalk and other pain neurobehavior were analyzed via repeated-measure anlysis of variance (ANOVA) followed by Bonferroni’s multiple comparison method. Student’s *t*-test also was used to compare the experimental results among groups. A *p*-value less than 0.05 was considered significant. The statistical analyses were conducted using SPSS software version12.

## 5. Conclusions

In this study, we found that DLK was highly expressed in various nerve tissues from crushed nerve to brain. This high expression was correlated to the intensity of neuropathic pain. The intrathecal injection of DLK shRNA attenuated the time course of neuropathic pain either in histomorphology or neurobehavior. It seems that manipulation of DLK has the potential to treat neuropathic pain in human beings; it is not mandatory, but can be added to the manuscript if the discussion is unusually long or complex.

## Abbreviations

DLK	Dual leucine zipper kinase
shRNA	Short hairpin RNA
MAP3K	Mitogen-activated protein kinase kinase kinase
MAPK	Mitogen-activated protein kinase
ERK	Extracellular signal-regulated kinase
JNK	c-Jun N-terminal kinase
DRG	Dorsal root ganglion
